# Potential Cost Saving of Epoetin alfa in Elective Hip or Knee Surgery due to Reduction in Blood Transfusions and Their Side Effects: A Discrete-Event Simulation Model

**DOI:** 10.1371/journal.pone.0072949

**Published:** 2013-09-09

**Authors:** Jörg Tomeczkowski, Sean Stern, Alfred Müller, Christian von Heymann

**Affiliations:** 1 Department of Health Economics, Janssen-Cilag GmbH, Neuss, Germany; 2 United Biosource Corporation, Bethesda, Maryland, United States of America; 3 Analytic Services GmbH, München, Germany; 4 Department of Anesthesiology and Intensive Care Medicine, Charité-University Medicine Berlin, Campus Virchow-Klinikum, Berlin, Germany; Gentofte University Hospital, Denmark

## Abstract

**Objectives:**

Transfusion of allogeneic blood is still common in orthopedic surgery. This analysis evaluates from the perspective of a German hospital the potential cost savings of Epoetin alfa (EPO) compared to predonated autologous blood transfusions or to a nobloodconservationstrategy (allogeneic blood transfusion strategy)during elective hip and knee replacement surgery.

**Methods:**

Individual patients (N = 50,000) were simulated based on data from controlled trials, the German DRG institute (InEK) and various publications and entered into a stochastic model (Monte-Carlo) of three treatment arms: EPO, preoperative autologous donation and nobloodconservationstrategy. All three strategies lead to a different risk for an allogeneic blood transfusion. The model focused on the costs and events of the three different procedures. The costs were obtained from clinical trial databases, the German DRG system, patient records and medical publications: transfusion (allogeneic red blood cells: €320/unit and autologous red blood cells: €250/unit), pneumonia treatment (€5,000), and length of stay (€300/day). Probabilistic sensitivity analyses were performed to determine which factors had an influence on the model's clinical and cost outcomes.

**Results:**

At acquisition costs of €200/40,000 IU EPO is cost saving compared to autologous blood donation, and cost-effective compared to a nobloodconservationstrategy. The results were most sensitive to the cost of EPO, blood units and hospital days.

**Conclusions:**

EPO might become an attractive blood conservation strategy for anemic patients at reasonable costs due to the reduction in allogeneic blood transfusions, in the modeled incidence of transfusion-associated pneumonia andthe prolongedlength of stay.

## Introduction/Background

Orthopedic surgery of the knee and hip continues to result in allogeneic blood transfusion [1] even as various blood conservation strategies have been instituted [2], [3] and thus largely remains an unmet medical need. Recently published guidelines [4] have outlined a management strategy aimed at detecting, evaluating and managing preoperative anemia in an effort to decrease the frequency of allogeneic blood transfusion and subsequently improve patient outcomes. The guidelines state that the most effective strategy to avoid postoperative anemia and blood transfusions, both of which are associated with morbidity and mortality [5], is to ‘identify and correct preoperative anemia whenever possible.’

Allogeneic blood transfusion not only poses a burden to the health of patients but also to society as a whole through additional costs and blood shortages. Recent estimates of the German blood supply indicate that this may not be an issue because 4,786,732 red blood cell units were manufactured and 4,311,110 red blood cell units were consumed in the year 2011 [9]. Predictive models created with the incorporation of demographic changes have highlighted how the burden of blood demand and supply will change over time [10], [11]. One such study predicted a 47% shortfall for in-hospital patients needing transfusions by 2020 for the German federal state Mecklenburg-Pomerania [12], [13]. In an effort to curtail demand for blood transfusions, the World Health Organization (WHO) has issued several recommendations, one of which is the use of erythropoietin [14].

Epoetin alfa (EPO) has been available and approved for preoperative use in hip and knee arthroplasty since 1996 [15]; however, it has not been widely adopted. In a large European survey, 1,239 patients of the 3,996 patients investigated were anemic (31%) with only 122 treated with EPO [16] which corresponds to 3% EPO usage in all patients (Of note, the EPO usage for Germany was suspected to be much lower). In contrast, for the 343,549 patients undergoing orthopedic surgery in Germany in 2010, allogeneic blood transfusion was applied to 75,841 (22%) and autologous blood to 9,298 (2.7%) of 23,400 (6.8%) who donated autologous blood (Procedures were taken from the German DRG system comprising 80% of German hospitals [17]and adjusted to the full population with all arthroplasty procedures by the Destatis dataset [18]).

While there are many possible reasons for EPO's inability to be adopted in clinical practice, the main reason appears to be its cost per regimen, which was originally around $2,250 [19] for the average 70 kg patient. When first brought to market, the cost per regimen (three weekly injections administered subcutaneously at 600 IU·kg^−1^ each) for the average 70 kg patient was approximately $268 per 20,000 IU. Multiple cost-effectiveness studies, using a similar dosing regimen and cost, found EPO to be well outside the boundaries of cost-effectiveness, with a cost per QALY greater than $1,000,000 in both cases [20], [21]. A suggested approach to reaching cost-effectiveness or possibly cost savings is to bring the regimen dose to an optimal level that trades off a smaller dosage size for a less effective regimen in preventing allogeneic blood transfusions [15]. One such study, Rosencheretal. 2005, demonstrated epoetinalfa's ability to raise hemoglobin (Hb) prior to surgery with only two injections of 40,000 IU [22], [23]. This regimen may reflect current European clinical practice and thus newer cost analyses of EPO are warranted. Green et al. 2010 [24] performed a cost minimization analysis, comparing the total costs of an allogeneic blood transfusion strategy against an autologous and allogeneic blood transfusion strategy for 161 primary total hip arthroplasty (THA) and 195 total knee arthroplasty (TKA) patients from a single center in the United States of America (USA). The EPO strategy model predicted costs similar to the autologous and allogeneic transfusion strategies at USA prices of $391 for a unit of autologous blood, $541 for 1 U of allogeneic blood and $489 for 40,000 IU of epoetin alfa. Martinez et al. 2007 [25]evaluated the costs associated with a blood saving algorithm in hip and knee arthroplasty for Europe. They found that the costs for EPO and its administration (€456/40,000 IU) were offset by a reduction in hospital transfusion costs (€278/unit autologous blood and €172/unit allogeneic blood). With the risk for blood transfusion following arthroplasty being highly dependent on the baseline characteristics of the patients, the relatively small sample size of the studies and the single center design may bias the results. To account for this variation and more accurately assess the costs, an individual patient simulation model was created that incorporates all knee and hip arthroplasty patients in Germany as well as individual characteristics specific to that population that could drive the need for allogeneic blood transfusion. It was the purpose of this investigation to assess with the above described patient simulation model whether the cost-effectiveness of Epoetin alfa (EPO) as compared to preoperative autologous donation (PAD) and a no blood conservation strategy using allogeneic blood transfusion only (ABT) is already reached or will be reached with further declining acquisition costs.

## Methods

### Model Design

The model compares 3 different strategies for a German population: EPO 40,000 IU per injection as recommended in the label with a regimen stratified to the pre-operative Hb, pre-surgery autologous blood donation (PAD), and a no blood conservation strategy (allogeneic blood transfusion strategy). The model assesses the costs that are associated with the entire episode of care for hip or knee arthroplasty from the hospital perspective, which limits the events and associated costs to those immediately associated with the inpatient stay. In order to provide a more robust analysis, the model randomly creates and records 50,000 individual patients from Germany's entire hip and knee arthroplasty population [26] using Microsoft Excel® in conjunction with utilizing Crystal Ball®'s ability to record iterative results. The model is provided as supporting information in Table S1.

As previously stated, the risk of transfusion during arthroplasty is highly dependent on the preoperative characteristics of the patient; therefore, this model takes into account variability in characteristics at the individual level. Through a process described below, a patient is created and assigned a set of characteristics that are drawn randomly from a set of age- and sex-specific distributions for each specific characteristic. Each patient is then replicated so that each clone is assigned to one of the three blood management strategies: nobloodconservation using allogeneic blood transfusion only (ABT), pre-surgery autologous blood donation (PAD), and pre-surgery EPO administration (EPO). All three procedures can lead to an allogeneic blood transfusion but with a different relative risk. Based on the patient characteristics and the blood management strategy, the model simulates that patient's surgery episode of care and assigns costs accordingly.

#### Patient Creation

The model begins by creating an individual patient and first assigns a patient's diagnosis-related group (DRG) code, age band and gender [26]simultaneously. These characteristics are generated by randomly indexing a table populated with data from German hip and knee arthroplasty procedures for 2005. Specifying the procedure at the DRG level, rather than merely discriminating between hip or knee arthroplasty, accounts for the fact that there are procedural differences that result in varying length of stay, surgical complexity and populations that typically have that specific procedure. Since the referenced German hip and knee table assigns a given patient an age band, the model further assigns a specific age within that band to the patient since other characteristics are modified by a specific age, not an age band.

Based on the patient's assigned age and gender, physiological parameters that are involved with determining the magnitude and relative severity of blood loss that occurs during hip or knee arthroplasty are also assigned to the patient. The model uses a normal distribution to determine a person's weight based on the age band and gender that individual was previously assigned [18]. Their weight is then used to determine estimated blood volume (EBV) by multiplying weight by 65 [25]. Finally, a baseline Hb level is assigned for the patient based on a gender-specific Weibull distribution which is fitted to available data from a chart review of knee and hip arthroplasty patients from RKU Universitäts- und Rehabilitationskliniken in Ulm, Germany (Text S1).

Patients' characteristics are further defined by aspects that independently increase the magnitude of blood loss a patient will experience during their hip or knee arthroplasty. Based on the DRG a patient is assigned, they are at risk of having an ASA score ≥III which results in a 5% increase in the amount of blood loss [27]. The ASA score is a physical status classification system that was adopted by the American Society of Anesthesiologists whereby a patient with an ASA score ≥III at the very least has severe systemic disease [28] and, accordingly, a high operative risk. Old age (≥75 years old) also increases the amount of blood loss by 5% [29]. Each DRG assigned has a risk of requiring a revision surgery [26], [29], which increases the expected amount of blood loss by 25% [30].

Since the model takes data from multiple sources, covariance between parameters that increase blood loss in patients could not be determined; therefore, sensitivity analyses were conducted to address this by setting an upper bound (1) and lower bound (0) for the degree of correlation. This was done by setting the increase in blood loss for revision surgery, ASA score ≥III and old age to 0%; however, the results from this analysis were not markedly different from the base case, indicating that the results were driven by other factors.

Patients also may be assigned the condition of rheumatoid arthritis based on a knee or hip specific risk. This characteristic does not affect the amount of blood loss that occurs during surgery; instead, rheumatoid arthritis worsens the effectiveness of EPO [31] [32], if the patient is assigned to that blood management arm. Patients may also have a history of cardiac events, which increases the transfusion trigger (a pre-defined level of Hb prompting a blood transfusion if patients are at or below this level) by 1 g/dlHb [33].

**Figure 1:**
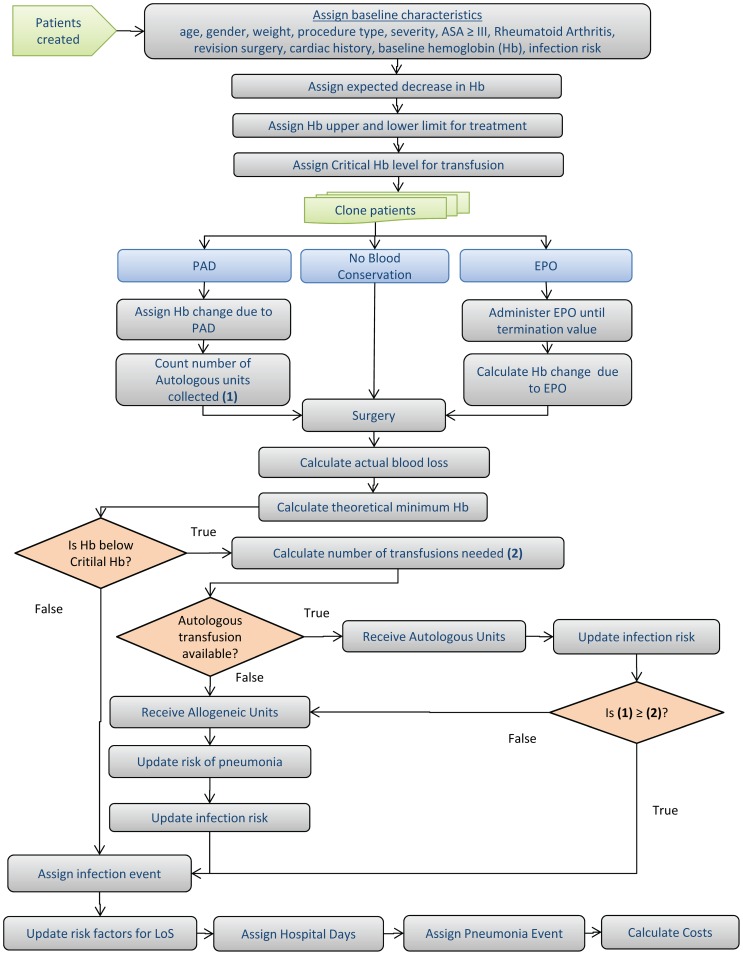
Algorithm of Patient Creation and of Modeled Blood Transfusion.

The final characteristic assigned to the patient is projected blood loss during the surgery. This is assigned by randomly drawing from a knee or hip specific distribution for blood loss [34]. This blood loss is increased beyond what it would normally be if the patient has any of three aforementioned complicating factors (ASA score ≥III, ≥75 years old, and revision surgery). Figure 1 demonstrates the interconnectedness of each of these aspects and their influence on Hb levels.

#### Patient allocation to blood management arms

With the patient's characteristics assigned, the model clones the patient so that the same patient is run through the aforementioned three blood management arms. All patients that lose blood during their knee or hip arthroplasty and meet or exceed the transfusion trigger receive as many units of allogeneic blood as needed to return to a level equal to or exceeding the trigger.

Patients under the PAD strategy have two units of blood donated prior to the surgery, which are then used when needed. If a patient needs more units of blood than have been donated, they must receive allogeneic blood transfusion. By donating blood prior to surgery, a patient's Hb levels are decreased at the time of surgery by a varying amount based on a distribution published by Stowell et al. [35].

When a patient is assigned to EPO, in the base case, they receive 40,000 IU injections of epoetin alfa until their Hb reaches a termination of 13.3 g/dl (40% Hematocrit). Each injection increases the patient's Hb level by an amount randomly determined from a distribution of Hb increase from EPO injections in a randomized study reported by Rosencher et al. [22]. In this study the patients received up to 3 injections (2 injections on average) until an Hb level of 13.3 g/dl was reached. No injection was given on the day of surgery. The protocol published by Weber et al. [31], in which patients received Epoetin alfa until their Hb reached a termination of 15 g/dl or a maximum of 4 injections (SMPC EPREX/ERYPO), was used as a sensitivity analysis. In this protocol patients received 3.5 injections on average; 1 injection was given on the day of surgery, which could not have any effect on the patient's Hb level on the day of surgery and therefore had no effect on blood loss on the day of surgery. An increase in Hb was taken from the EPO arm but not as a difference to the control arm because in the control arm autologous blood donation could be applied, leading to a lower Hb at the day of surgery.

With each cloned patient now assigned a blood management strategy, their baseline Hb is adjusted accordingly based on that strategy. The patient then undergoes the procedure previously assigned to them and incurs the predetermined amount of blood loss based on relevant assigned characteristics. The post-surgery Hb level is calculated by subtracting the blood loss from the surgery (in terms of Hb) from the adjusted baseline Hb. This post-surgery Hb is compared to one of three transfusion triggers that were chosen [Hb = 8.5 (base case), Hb = 9.0 (liberal), or Hb = 8.0 (strict)] to determine if a transfusion is needed. This transfusion trigger is increased by 1 g/dl Hb if a patient has a history of cardiac issues; regardless of the transfusion trigger value is set to [33]. If transfusion is needed, the number of units of blood needed to meet or exceed the given transfusion trigger are administered to the patient (Figure 1).

#### Resource Utilization and Costs

Each patient who undergoes a transfusion accrues a cost for each unit of allogeneic blood transfused at a cost of €320 per unit [36]. All patients assigned to the PAD blood management strategy who are eligible to receive autologous blood transfusion incur a cost of €500 for the two units of blood donated and stored [37]. Cost calculations consider major process steps, staff, and consumables to provide red blood cell (RBC) transfusions to surgical patients, including usage frequencies and direct and indirect overhead costs, the latter of which were 3.2– to 4.8-fold higher than blood product acquisition costs [38]. Patients assigned to EPO incur a cost of €200 per 40,000 IU dose administered. The cost for Epoetin alfa incorporates an average rebate on the list price of €375 per 40,000 IU, which is usually negotiated with hospitals in Germany. Sensitivity analyses were conducted around all costs (±25%).

The model considers other resource utilization that is incrementally affected by a blood transfusion, compared to not having a blood transfusion, while in relation to its impact on the length of stay of a patient undergoing hip or knee arthroplasty. Each patient has a projected length of stay based on the DRG they are assigned, which is then adjusted upwards by transfusion-related events. First, patients that have a blood transfusion have their length of stay increased by 20% [39]. Second, while all patients are at risk of infection while in the hospital undergoing arthroplasty, patients that receive an allogeneic or autologous blood transfusion are at higher risk of infection, which results in an increased length of stay of 60% [40]. If a patient has both a transfusion and infection during their hospital stay, the patient's length of stay is increased by 90% [41] compared to the baseline. The length of stay for a patient is also adjusted to account for their age. Patients accrue a cost of €300 for each day they spend in the hospital.

Patients undergoing hip or knee arthroplasty also face a background risk of pneumonia that is increased by a factor of 2 (1.6% in transfused versus 0.8% in non-transfused patients) when a patient undergoes a blood transfusion [1]. The model assigns this cost (€5,000) [42]on a per-event basis rather than indirectly increasing costs by increasing the patient's length of stay.

A table containing all the model's inputs can be found in Table S1 and in Text S1.

#### Analyses Conducted

The model produces results that are stratified by the patient's baseline Hb levels prior to the assignment of the blood management strategy in increments of 0.5 g/dl Hb over a range starting from 10 g/dl to 13 g/dl. Since there are variations in transfusion triggers [43], the base case parameters and population were used for three separate analyses with transfusion triggers at Hb 8.5 g/dl (base case), 8.0 g/dl (strict) and 9.0 g/dl(liberal). Sensitivity analyses were also conducted to examine which parameters had the most influence on model results including the amount of blood loss in terms of Hb during both hip and knee arthroplasty, which was varied to values from other sources [44]. Based on a review of other sources, the base case blood loss value for hip and knee arthroplasty [34], [45]was higher than other sources found and therefore the sensitivity analyses around blood loss was referred to ‘blood loss low’ and ‘blood loss medium’. Focusing on a subgroup with expected high blood loss, one scenario was calculated for patients with revision surgery. Another key parameter to the model was the length of stay (LOS) increase due to allogeneic blood transfusion, which was varied across its 95% CI (mean  = 20%; 11%–29%) [44]. All sensitivity analyses were conducted with a trigger of 8.5 g/dland Hb levels between 10.0–13.0 g/dl for ABT vs. EPO and 11.0–13.0 g/dl for PAD vs. EPO because PAD is not indicated for patients with preoperative Hb levels below 11.0 g/dl. All costs were varied individually by ±25% and certain parameter values were changed to alternative sources (Table 1).

**Table 1:** Model input in base case configuration and their sensitivity variables.

Parameter	Value	Parameter values / % changes for Sensitivity Analyses				References
**Transfusion trigger [g/dl]**	8.5	8.0	9.0			
**Surgery procedure**	All	Revision				
**EPO 40.000 IU injections [n]**	2*	3.5**				[22], [31]
**EPO effect on preop. Hb [g/dl]**	+2.0*	+2.1**				[22], [31]
**PAD effect on preop. Hb [g/dl]**	−1.2	−1.16	−1.05	−.8	−0.67	[31], [34], [35], [59], [60]
**Blood loss hip [Hb g/dl]**	−3.3	−3.1	−2.8	−2.1		[31], [45]
**Blood loss knee [Hb g/dl]**	−3.5	−2.9	−2.5	−1.9		[31], [45]
**Cost EPO 4×40.000 IU [€]**	800	+25%	−25%			
**Cost RBC per unit [€]**	320	+25%	−25%			
**Cost PAD per unit [€]**	250	+25%	−25%			
**Length of stay transfused patients**	+20%	+5%	+11%	+29%	+35%	
**Rate of Pneumonia in transfused patients**	2x					[1]
**Cost total [€]**		+25%	−25%			

* Up to 3 injections until Hb of 13 g/dl was reached; no injection on the day of surgery. ** Up to 4 injections not exceeding 15 g/dl (3.5 injections on average); 1 injection was given at day of surgery which could not have any effect on the Hb at the day of surgery. preop.  =  preoperative; Hb  =  Hemoglobin.

## Results

The model simulated 50,000 patients with hip and knee arthroplasty procedures from a German data set. Of those 50,000 patients, 11,602 patients fit the baseline Hb band of 10.0 to 13.0 g/dl, chosen for this analysis because patients with Hb levels within this band would benefit from EPO. Base case results for EPO vs. ABT and PAD are presented in Table 2. The table presents clinical, resource and cost results that are stratified by first visit preoperative Hb bands of 0.5 starting at 10.0 g/dl for ABT vs. EPO and 11.0 g/dl for PAD vs. EPO (PAD would not be an option for patients with baseline Hb below 11.0 g/dl). For ABT vs. EPO, the EPO strategy is cost-saving for all Hb bands, and EPO also provides significant cost savings in comparison to PAD for every Hb band applicable. The main driver of model results is the frequency of transfusions (both autologous and allogeneic) as these increase costs due to the transfusion costs themselves, the extended length of stay for patients, and pneumonia costs.

**Table 2:** Model results for patients with different preoperative Hb-values (grouped in classes from 10 – 13 g/dl).

Parameter/	10.0–10.5	10.5–11.0		11.0–11.5	11.5–12.0	12.0–12.5	12.5–13.0
Preoperative Hb group [g/dl]							
Simulated cases [n]	354	644		1091	1884	3057	4572
	Mean (SE; SD)	Mean (SE; SD)		Mean (SE; SD)	Mean (SE; SD)	Mean (SE; SD)	Mean (SE; SD)
**Baseline Characteristics**							
Percentage of patients (50,000) [%]	0.71 (0.1; 8.4)	1.29 (0.1; 11.3)		2.18 (0.1; 14.6)	3.77 (0.2; 19.0)	6.11 (0.2; 24.0)	9.14 (0.3; 28.8)
Female [%]	87.9 (1.7; 32.7)	84.3 (1.4; 36.4)		83.5 (1.1; 37.1)	81.2 (0.9; 39.1)	81.9 (0.7; 38.5)	81.1 (0.6; 39.1)
Age [years]	71.3 (0.52; 9.8)	71.9 (0.37; 9.4)		71.1 (0.31; 10.3)	71.3 (0.23; 10.1)	71.4 (0.18; 10.1)	71.1 (0.15; 10.3)
Revision Surgery [%]	13.0 (1.8; 33.6)	10.4 (1.2; 30.5)		10.4 (0.9; 30.5)	10.4 (0.7; 30.5)	9.1 (0.5; 28.8)	10.4 (0.5; 30.6)
Hip Surgery [%]	56.5 (2.6; 49.6)	56.2 (2.0; 49.6)		58.2 (1.5; 49.3)	58.0 (1.1; 49.4)	58.7 (0.9; 49.2)	59.1 (0.7; 49.2)
ASA ≥III [%]	40.1 (2.6; 49.0)	41.0 (1.9; 49.2)		41.9 (1.5; 49.3)	40.7 (1.1.; 49.1)	40.3 (0.9; 49.0)	40.3 (0.7; 49.0)
Cardiac Disease History [%]	15.5 (1.9; 36.2)	13.2 (1.3; 33.8)		13.9 (1.0; 43.6)	13.8 (0.8; 34.5)	14.3 (0.6; 35.0)	15.2 (0.5; 35.9)
Hb base [g/dl]	10.27 (0.008; 0.15)	10.78 (0.006; 0.14)		11.27 (0.004; 0.14)	11.77 (0.003; 0.14)	12.27 (0.003; 0.14)	12.77 (0.002; 0.14)
**ABT(allogeneic blood transfusion only**)							
*Clinical and Resource Utilization*							
Transfused [%]	94.4 (1.2; 23.1)	88.8 (1.2; 31.5)		77.3 (1.3; 41.9)	59.3 (1.1; 49.1)	43.3 (0.9; 49.6)	28.4 (0.7; 45.1)
Mean units per transfusion [n]	2.5 (0.07; 1.3)	2.1 (0.05; 1.1)		1.8 (0.04; 1.0)	1.6 (0.03; 0.9)	1.5 (0.02; 0.8)	1.4 (0.02; 0.7)
Mean LOS [days]	16.2 (0.25; 4.6)	15.9 (0.18; 4.6)		15.7 (0.14; 4.7)	15.1 (0.11; 4.6)	14.6 (0.08; 4.4)	14.2 (0.06; 4.2)
*Costs*							
Transfusion cost [€]	751(24; 454)	593(16; 396)		444 (11; 376)	306 (8; 336)	203 (5; 285)	123 (3; 227)
LOS [€]	4,857 (74; 1,395)	4,762 (54; 1,370)		4,699 (42; 1,400)	4,522 (32; 1,839)	4,367 (24; 1,334)	4,249 (19; 1,268)
Infection (Pneumonia) [€]	113 (39; 743)	47 (19; 480)		87 (20; 654)	48 (11; 486)	77 (11; 615)	47 (7; 483)
Total [€]	5,721 (89; 1669)	5,402 (62; 1,572)		5,231 (51; 1,676)	4,875 (36; 1,563)	4,646 (29; 1,590)	4,419 (21; 1,426)
**PAD**							
*Clinical and Resource Utilization*							
Hb decrease [g/dl]	-	-		−1.18 (0.03; 0.83)	−1.17 (0.03; 0.84)	−1.16 (0.02; 0.83)	−1.17 (0.02; 0.83)
Transfused (autologous) [%]	-	-		88.9 (1.1; 31.4)	81.3 (1.2; 39.0)	73.1 (1.2; 44.3)	62.5 (1.3; 48.4)
Mean units per transfusion [n]	-	-		1.9 (0.01; 0.3)	1.8 (0.01; 0.4)	1.7 (0.01; 0.4)	1.7 (0.01; 0.5)
Transfused (allogeneic) [%]	-	-		68.8(1.6; 4.63)	53.2 (1.5; 49.9)	39.1 (1.3; 48.8)	27.0 (1.2; 44.4)
Mean units per transfusion [n]	-	-		2.3 (0.05; 1.5)	2.1 (0.04; 1.4)	1.8 (0.02; 1.1)	1.7 (0.03; 1.1)
Infection [%]	-	-		3.1 (0.6; 17.4)	4.3 (0.6; 20.3)	3.6 (0.5; 18.6)	3.8 (0.5; 19.1)
Mean LOS	-	-		16.1 (0.16; 4.7)	15.8 (0.14; 4.8)	15.4 (0.12; 4.5)	15.1 (0.12; 4.5)
*Costs*							
Blood collection + Auto. Transf. [€]	-	-		500 (0; 0)	500 (0; 0)	500 (0; 0)	500 (0; 0)
Transfusion cost (allogeneic) [€]	-	-		510 (18; 517)	356 (14; 467)	228 (10; 366)	145 (8; 297)
LOS [€]	-	-		4,816 (49; 1,413)	4,727 (43; 1,434)	4,619 (37; 1,363)	4,531 (37; 1,344)
Infection (Pneumonia) [€]	-	-		82 (173; 5,019)	50 (148; 4,940)	79 (132; 4,815)	44 (131; 4,726)
Total [€]	-	-		5,909 (58; 1,686)	5,634 (48; 1,604)	5,426 (44; 1,598)	5,220 (41; 1,482)
**EPO**							
*Clinical and Resource Utilization*							
HB increase [g/dl]	2.21 (0.07; 1.23)	2.25 (0.05; 1.32)		2.34 (0.04; 1.45)	2.35 (0.09; 1.39)	2.20 (0.02; 1.20)	2.05 (0.02; 1.11)
Transfused [%]	37.3 (2.6; 48.4)	30.4 (1.8; 46)		22.1 (1.3; 41.5)	13.5 (0.8; 34.2)	9.4 (0.5; 29.1)	4.9 (0.3; 21.5)
Mean units per transfusion [n]	1.8 (0.09; 1.1)	1.5 (0.06; 0.9)		1.5 (0.05; 0.7)	1.4 (0.04; 0.7)	1.4 (0.04; 0.7)	1.3 (0.05; 0.7)
Infection [%]	2.8 (0.9; 16.6)	2.2 (0.6; 14.6)		2.6 (0.5; 15.8)	2.7 (0.4; 16.2)	2.2 (0.3; 14.6)	2.4 (0.2; 15.4)
Mean LOS [days]	14.6 (0.2; 4.6)	14.4 (0.2; 4.4)		14.2 (0.1; 4.3)	13.8 (0.1; 4.2)	13.6 (0.1; 4.0)	13.5 (0.1; 3.9)
*Costs*							
EPO treatment [€]	559 (8; 144)		553 (5; 133)	525 (4; 139)	462 (4; 168)	383 (3; 178)	285 (2; 155)
Transfusion cost [€]	218 (19; 350)		147 (11; 273)	104 (7; 225)	60 (4; 173)	41 (3; 148)	21 (2; 104)
LOS [€]	4,393 (73; 1,374)		4,306 (52; 1,330)	4,256 (39; 1,292)	4,154 (29; 1,258)	4,093 (22; 1,202)	4,060 (17; 1,158)
Infection (Pneumonia) [€]	42 (245; 4,603)		23 (178; 4,507)	60 (135; 4,447)	37 (100; 4,340)	62 (877; 4,265)	37 (62; 4,222)
Total [€]	5,212 (84; 1,572)		5,030 (58; 1,474)	4,945 (45; 1,476)	4,713 (32; 1,370)	4,580 (25; 1,376)	4,403 (19; 1,255)
*ABT vs. EPO (Incremental Total Cost)* [€]	**509** (**46; 870**)		**372** (**29; 724**)	**286** (**24; 802**)	**162** (**17; 739**)	**67** (**13; 731**)	**16** (**9; 629**)
*PAD vs. EPO (Incremental Total Cost)* [€]	-		-	**964** (**24; 787**)	**921** (**17; 750**)	**846** (**13; 746**)	**817** (**10; 653**)

SE  =  Standard Error; SD  =  Standard Deviation; ASA  =  American Society of Anesthesiologists physical status classification; EPO  =  epoetin; LOS  =  Length of stay; PAD  =  preoperative autologous blood donation; ABT  =  allogeneic blood transfusion only; Auto. Transf.  =  Autologous Transfusion.

**Figure 2:**
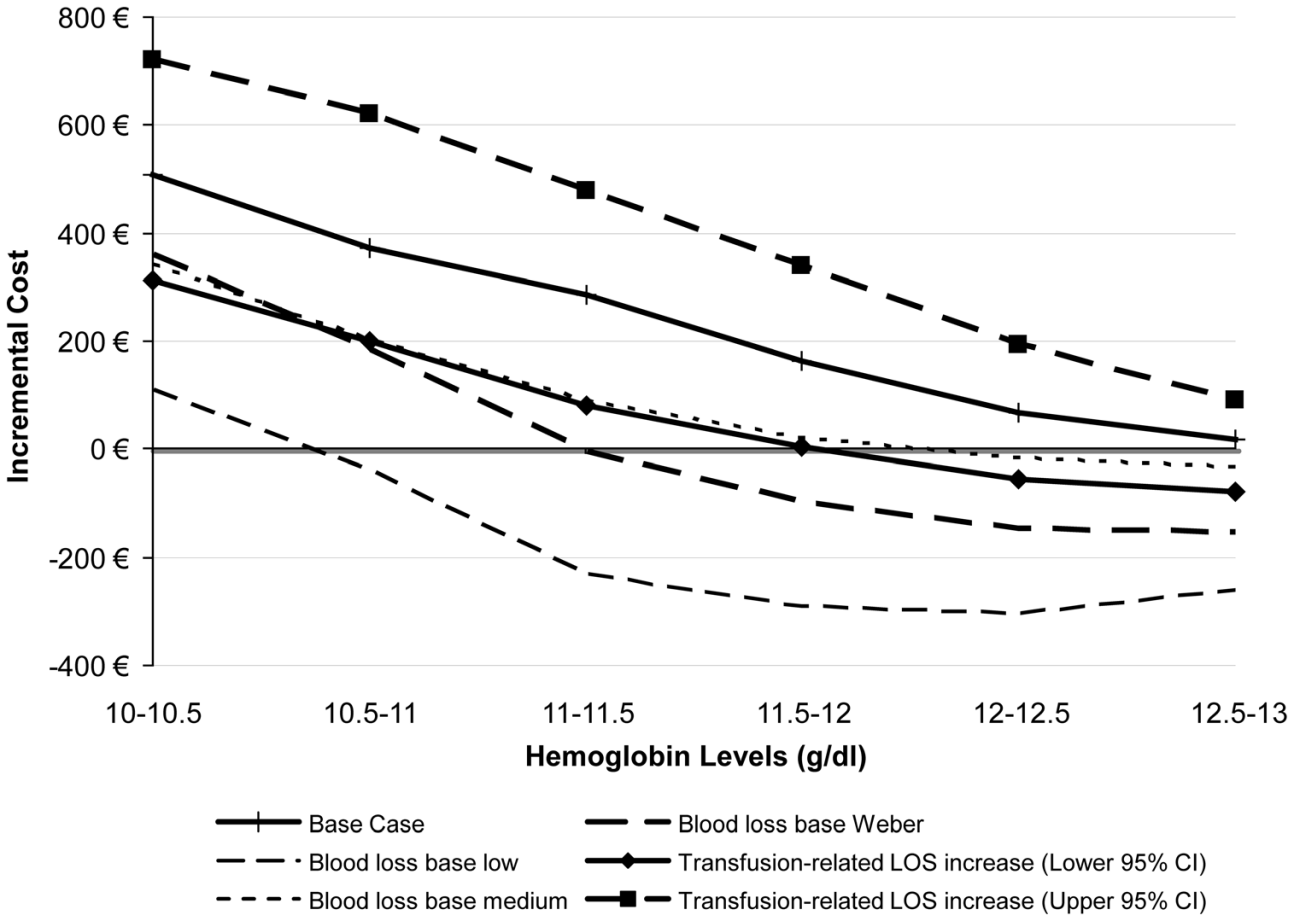
Incremental costs of ABT vs. EPO strategy – Sensitivity Analyses: Most Influential Parameters (Factor 1.11 and 1.29 are referring to lower and upper 95% Confidence interval).

**Figure 3:**
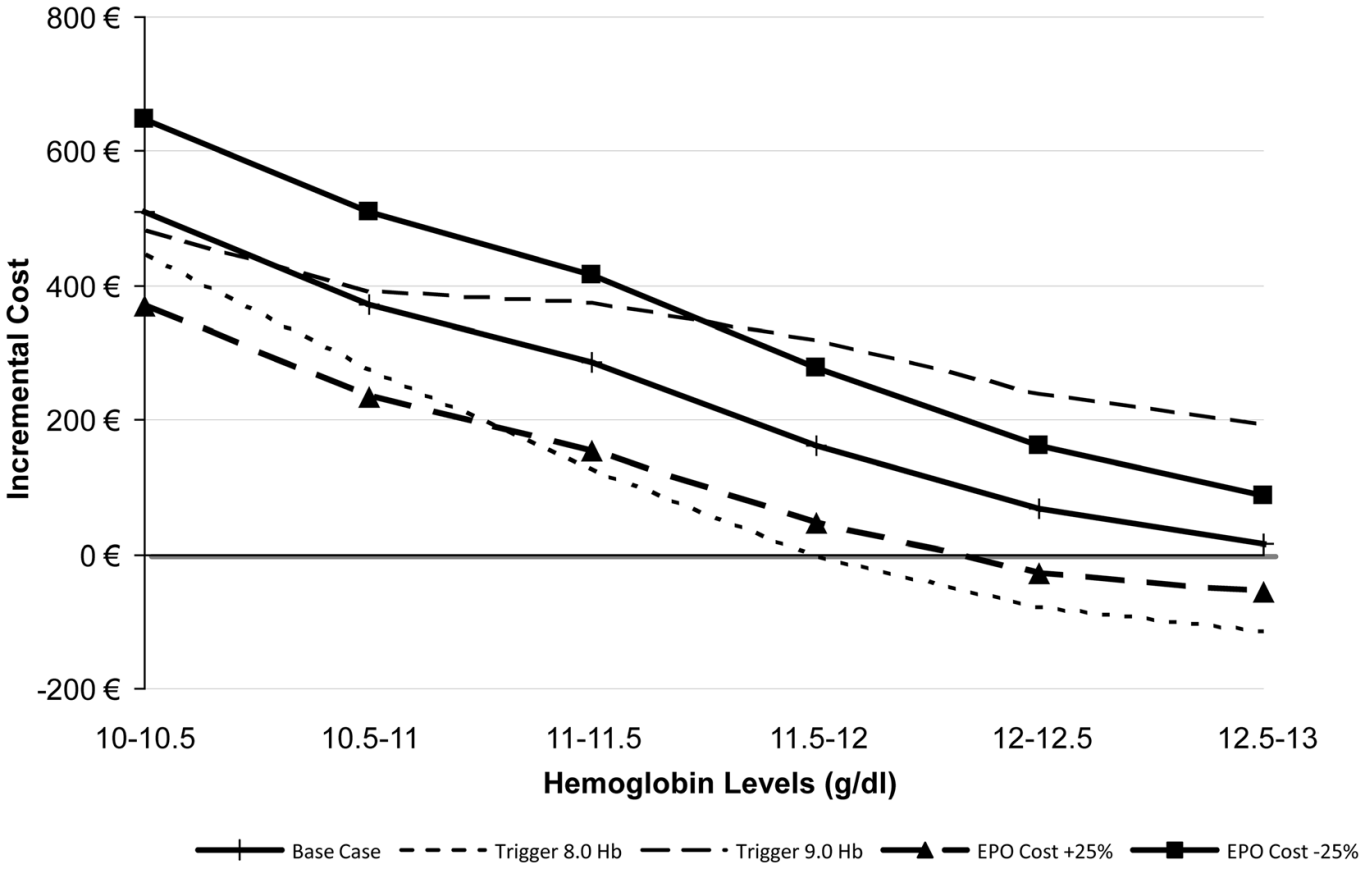
ABT vs. EPO – Sensitivity Analyses: Other Influential Parameters.

**Figure 4:**
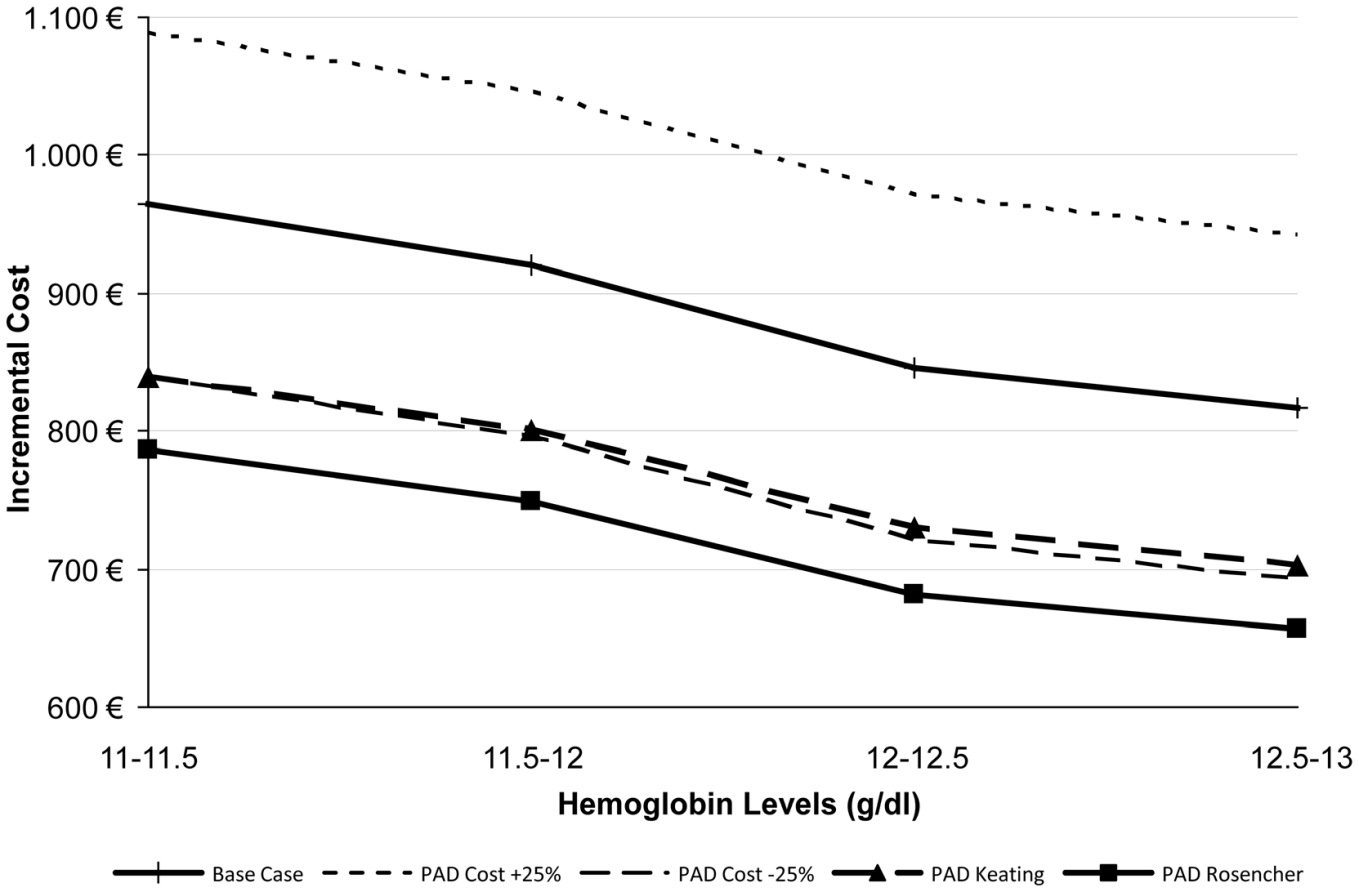
PAD vs. EPO – Sensitivity Analyses: PAD Parameters.

**Figure 5:**
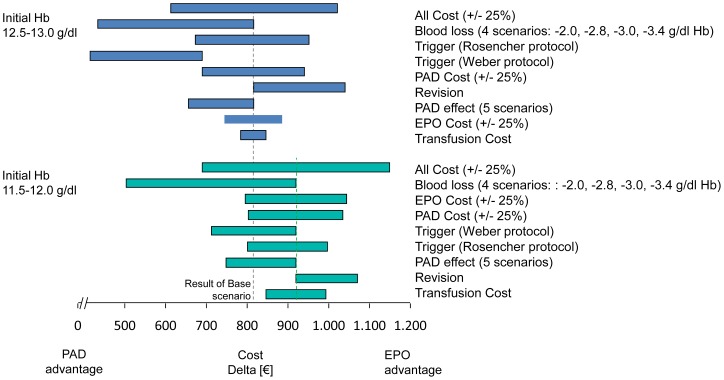
Sensitivity analysis –ABT vs. EPO by preoperative Hb level subgroups.

**Figure 6:**
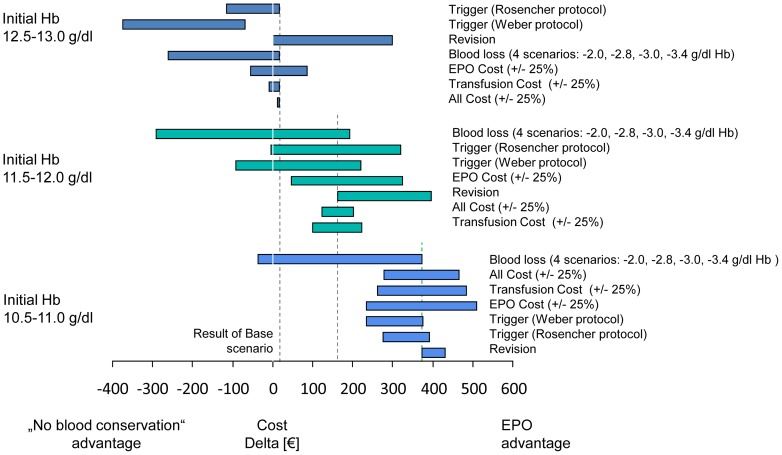
Sensitivity analysis –PAD vs. EPO by preoperative Hb level subgroups.

Figure 2 visually presents the parameters that have the greatest impact on model results over each Hb band for ABT vs. EPO. The model is very sensitive to the increase in LOS due to transfusion (both above and below the base case value) when the patient's baseline Hb is between 10–11.5 g/dl; however, its impact diminishes when the patient's baseline Hb is higher. Another parameter with a large impact on model results is the amount of blood loss a patient has during their arthroplasty; as the amount of blood loss is reduced, the cost differential between the blood management strategies shrinks dramatically. From a clinical point of view, these results are both consistent with the design of the model because higher baseline Hb results in fewer transfusions, and the amount of blood lost impacts whether the patient undergoes transfusion and incurs the associated costs (listed above). Figure 3 presents other sensitivity analyses surrounding other parameters with a large impact on model results for ABT vs. EPO and Figure 4 presents parameters examined specifically for PAD vs. EPO. Figure 5 and 6 present all of the results from the analyses that were conducted to examine the model results' sensitivity to certain parameter values (see Text S1 for tabled results).

## Discussion

This economic model evaluates the usage of EPO compared to PAD and a no blood conservation strategy (ABT) of a clinical patient blood management program in order to avoid allogeneic blood transfusion in hip or knee arthroplasty surgery. At acquisition costs of €200/40,000 IU, EPO is cost saving when compared to preoperative autologous blood donation (PAD), and cost-effective compared to allogeneic blood transfusion (no blood conservation strategy) for patients suffering from anemia with an Hb level in the range of 10 – 13 g/dl. At acquisition costs of €375/40,000 IU (current list price) EPO is cost saving compared to PAD. The results were most sensitive to the cost of EPO, blood units and hospital days.

Blood management programs in observational studies [25], [39] and in one cluster-randomized trial [40], [46], [47], respectively, from Europe and Canada have shown encouraging results. In these programs the widespread use of PAD was common, as it still is in Germany. The studies found that a systematic approach to optimize patients' red cell mass and to limit Hb loss perioperatively was associated with lower allogeneic and autologous transfusion rates, shorter LOS, and a reduction in re-attendance after elective arthroplasty [39], [40], [46], [47]. Although these results are encouraging, blood management programs including the correction of anemia with EPO and/or iron are not frequently used in orthopedic surgery in Germany. The reasons for not treating anemic patients could include the perceived high costs of EPO, the need for a multidisciplinary approach to identify and to treat patients, and the time needed for anemia treatment, which postpones hospital admissions and possibly interferes with acuity and urgency of the orthopedic procedure. Since the donation of autologous blood, like the administration of EPO, is a complex and multidisciplinary approach to treatment, we therefore compared in our model the costs for EPO also with the costs for autologous donation.

With our model we can demonstrate from the perspective of a German hospital that the costs of EPO should not be a barrier to implementing blood management programs for anemic patients. The model input configuration for the base case and sensitivity analyses gives a hospital the opportunity to check the cost effectiveness for EPO in their specific situation for different levels of anemia.

The Network for Advancement of Transfusion Alternatives (NATA) has published blood management guidelines for patients undergoing orthopedic surgery. NATA recommends that gender-specific WHO criteria for anemia diagnosis also be used as treatment thresholds. We used these thresholds in our model, but transfusion triggers were not gender-specific. In our model, women and men lost similar amounts of Hb (and hence proportionally similar blood volumes) perioperatively. However, women had lower preoperative Hb values and subsequently had higher transfusion rates. Gender-specific treatment thresholds may therefore expose women to disproportionate risk of transfusion and possible consequent morbidity.

In our model, hip arthroplasty was more likely to lead to allogeneic blood transfusions than knee arthroplasty. This is consistent with the results published by Pierson et al. [45] and might be explained by the different age of patients. Pierson showed that patients undergoing knee arthroplasty were on average 69 years old while patients undergoing hip arthroplasty were on average 63 years old.

NATA also recommends that clinicians assess the initial Hb level as close as possible to 28 days before surgery and that they correct the Hb level according to the cause of anemia [4]. The cause of anemia in patients undergoing elective orthopedic surgery is in about 50% of cases anemia of chronic disease (ACD), for which EPO is approved and in about 25% of cases an iron deficiency. In the rest of patients the cause is Vitamin B12 or folic acid deficiency, or the reason is unknown [48]. If the patient was anemic, our model did not differentiate between anemia of chronic disease and iron deficiency anemia. Outcomes for anemic patients were modeled according to the outcomes from randomized clinical trials comparing EPO and PAD or EPO and usual care. In these trials anemia of chronic disease or iron deficiency was not differentiated and EPO was always administered with iron together as recommended. Overall, our baseline outcomes for transfusion [16] were comparable, but LOS was longer with those from elsewhere in Europe. The most frequent perioperative Hb value associated with transfusion was 8.0 – 8.9 g/dl in the OSTHEO study, a large multicentre investigation of transfusion for arthroplasty across Europe [16]. To be consistent with these results, we used a base case trigger of Hb 8.5 g/dl (determined based on patient charts), with sensitivity analyses exploring use of a stricter and a more liberal trigger, 8.0 g/dl and 9.0 g/dl respectively.

Most patients in the trials received oral iron, which is relatively cheap. Intravenously administered iron did not add to the effect of EPO for patients without iron deficiency in a randomized controlled trial [49], and therefore, it was not included in the model. Also, although thrombosis prophylaxis is recommended in the SMPC for Epoetin alfa [50], associated costs were not included because thrombosis prophylaxis is standard of care in Germany even without EPO therapy. The cost for EPO in the model is €200, with sensitivities of €250 and €150. Although the list price for EPO 40,000 IU is much higher in Germany, we assume a rebate for hospitals in the range of 50%. Since EPO is approved in order to avoid blood transfusions in orthopedic surgery, the hospital has to pay for EPO just as it pays for autologous blood donation, even if EPO is administered 3 weeks before surgery.

The model calculates an improvement in LOS if a transfusion could be avoided. A shorter LOS was observed in cluster randomized trials evaluating a blood conservation algorithm [46], [47]. Also Kotzé et al [39] observed improved measures of patient outcome, namely LOS and re-admission after implementing a blood management program.Delasotta et al. found a decreased LOS for patients treated with Epoetin alfa in knee arthroplasty [51] or hip arthroplasty [52]. Blood management programs were found generally to be cost-effective [24], [25]. In the program described by Kotzé et al. [39], the drug cost of the program was £16,695 over 8 months. Of this, £12,625 was offset by savings on the purchase of red cells, making the net ‘cash cost’ of implementing the algorithm £4,070 for a cohort of 281 patients. These measures further do not allow for nursing time, consumable equipment (e.g. blood giving sets vs. cheaper fluid sets), repeat blood tests, the treatment of any complications related to transfusion or the reduction in length of hospital stay that was by one day shorter in the patient blood management cohort. While these additional activity-based costs of transfusion are difficult to quantify, recent data indicate that they may be as much as four times the product cost [38]. Unlike Kotzé et al., we included these costs in our model. In addition, we also included costs for treating pneumonia associated with allogeneic blood transfusion, because in a retrospective study from 28,087 patients with hip or knee surgery it was shown that pneumonia was associated with blood transfusion [1] although the underlying mechanism for the higher risk of pneumonia has not been fully elucidated. Immunomodulatory changes [53], including an increased release of IL-10 and FasL after transfusion of red blood cells, have been described [54] and may contribute to immunosuppression possibly responsible for transfused patients being more susceptible to infections. Adverse outcomes after transfusion of red blood cells are also correlated with the storage time of the blood. This effect will be further investigated in two prospective controlled randomized clinical trials [55]. It is therefore likely that implementing a blood management program may be cost-saving by reducing infectious complications, leading to a shorter length of stay once total in-hospital costs are accounted for.

The model described has important weaknesses. The data for the outcomes length of stay and pneumonia are observational while the effectiveness of EPO, autologous blood donation and no blood conservation are taken from randomized control trials. In case of EPO the effectiveness on preoperative Hb was taken from randomized control trials [22], [31] and the reduction on transfusion rate from a meta-analysis consisting of 5 randomized trials [48]. We cannot comment on what the relative contributions of preoperative iron therapy and intraoperative measures were, since there are no published data from randomized clinical trials fitting the situation simulated. We did not model data on fluid management, complications other than pneumonia, or the reasons for health-care resource use after discharge, since these are not paid by the hospital. Furthermore, this model does not account for potential adverse events of EPO, e.g. thromboembolic complications. However, postoperative thromboprophylaxis (e.g. low molecular weight heparins) is used throughout Germany, as it is strongly recommended by current German guidelines [56] due to the high risk of deep venous thrombosis (DVT) or pulmonary embolism (PE) after hip or knee replacement surgery. The routine use of thromboprophylaxis may also prevent thromboembolic complications when EPO is administered as it has been recommended by the FDA [57]. Routine thromboprophylaxis might be the reason that there was no difference in the risk of developing thromboembolism between EPO and control groups in a meta-analysis with pooled results from 26 trials [58].

However, our model also has several strengths. We used a substantial baseline data set for a patient individual model from records subject to regular external audit. In contrast to a cohort model, where median or mean values are modeled, our model created patients with individual parameters. For all 50,000 patients modeled the outcomes were recorded for the three strategies investigated. Compared to a cohort model our used criteria ensured high specificity because the resultswere similar in size and ratio of hip to knee arthroplasty and procedure of surgical intervention to that which was reported to a national DRG-register.

Conclusions: The presented model showed that the EPO strategy is cost saving or cost-effective for the patients and procedures in base case configuration regardless of the comparator. Randomized trials are necessary to determine whether blood management programs improve patient outcomes other than transfusion rates, and what the optimal algorithm is in terms of cost and patient outcome. This will become even more important in the future, as upcoming blood shortages and increasing prices of red blood cells and transfusion-related complications will make a reasonable blood conservation strategy including EPO urgently needed.

## Supporting Information

Table S1 **Model input parameter and results.**
(XLS)

Text S1 **Details of methods, input data, costs and Weibull distribution of preoperative hemoglobin.**
(DOC)
