# Supplementary material for: Potential Cost Saving of Epoetin alfa in Elective Hip or Knee Surgery due to Reduction in Blood Transfusions and Their Side Effects: A Discrete-Event Simulation Model
Source: PLoS One. 2013 Sep 9;8(9):e72949. doi: 10.1371/journal.pone.0072949 (PMC3767728; doi:10.1371/journal.pone.0072949)
Supplement: Text S1 — Details of methods, input data, costs and Weibull distribution of preoperative hemoglobin. (DOC) [file pone.0072949.s002.doc]

**APPENDIX**

**POTENTIAL COST SAVING OF EPOETIN ALFA IN ELECTIVE HIP OR KNEE SURGERY DUE TO REDUCTION IN BLOOD TRANSFUSIONS AND ITS SIDE EFFECTS: DISCRETE-EVENT SIMULATION MODEL**

Jörg Tomeczkowski, Sean Stern, Alfred Müller, Christian von Heymann

Content

[A: Demographics and DRG code 2](#__RefHeading___Toc354256322)

[B: Risk Factors 2](#__RefHeading___Toc354256323)

[C: Physiological parameters 3](#__RefHeading___Toc354256324)

[C1: Pre-surgical blood management – Treatment with Epoetin Alfa 3](#__RefHeading___Toc354256325)

[C2: Pre-surgical blood management – Autologous Donation 4](#__RefHeading___Toc354256326)

[E: Blood loss and transfusion 4](#__RefHeading___Toc354256327)

[F: Care on ward, post-operative complications 5](#__RefHeading___Toc354256328)

[G: Unit costs 6](#__RefHeading___Toc354256329)

[Sensitivity Analyses to calculate the cost delta in Euro for EPO versus No Blood Conservation and EPO versus preoperative autologous blood donation 7](#__RefHeading___Toc354256330)

[Distribution selection for the initial Hemoglobin value (pre-surgery, first visit) 8](#__RefHeading___Toc354256331)

| Simulation phase | Distribution | Comment, Parameters | Source | Sensitivity Analysis |
| --- | --- | --- | --- | --- |
| A: Demographics and DRG code | | |  |  |
| Age band, gender, DRG code | random draw, empirical distribution | Triples of age band, gender and DRG code are drawn from a joint empirical distribution published for German DRG data | Institut für Entgeltwesen im Krankenhaus: DRG Browser für §21-Daten, Datenjahr 2005 ***(26)*** | No |
| Age | random draw, empirical distribution | 5-year age bands are distributed to age (whole number in years) using the gender-specific age pyramid published for Germany | Age distribution as of Dec 31st, 2006; Statistisches Bundesamt: Statistik Regional | No |
|  |  |  | www.destatits.de |  |
| B: Risk Factors | |  |  |  |
| ASA>=III | random draw, empirical distribution | ASA>=III proportions are approximated by high patient clinical complexity levels (published DRG-wise). PCCL levels 4, 3, and 25% of PCCL level 2 are assumed to be ASA >= III. The resulting proportion of ASA>=III is 39% (compared to 40% published by Rashiq et al) | Rashiq ***(29)***, table 1 | no |
| Age>=75 | computed |  |  |  |
| Rheumatoid Arthritis | random draw, empirical distribution | Hip surgery: 12.6% / Knee surgery: 36.1% | Source: Slappendel ***(32)***; table 2 | no |
| Revision surgery | random draw, empirical distribution | Implied by DRG code (4 out of 12 knee/hip arthroplasty codes). For all other codes, a marginal share of revisions (1%) was assumed. The total proportion of revision surgeries is 11.7% (compared to 11% published by Rashiq) | Institut für Entgeltwesen im Krankenhaus: DRG Browser für §21-Daten, Datenjahr 2005 ***(26)***; Rashiq ***(29)***, table 1 | One sensitivity case with revision procedures only |
| Cardiac history | Bernoulli | 15% prevalence with cardiac history in the relevant age bands. Assumption is conservative compared to the CHD prevalence data published by RKI as the results of the Bundesgesundheitssurvey 2006 (19.1% for women, 28.2% for men, self-reported) | Dr. Ute Ellert, Jutta Wirz, Dr. Thomas Ziese: Telefonischer Gesundheitssurvey des Robert Koch-Instituts (2. Welle). Deskriptiver Ergebnisbericht. Berlin (2006) http://edoc.rki.de/documents/ rki_fv/reJBwqKp45PiI/PDF/ 21r1eZ1NVL2AY_11.pdf | no |
|  |  |  |  |  |
| C: Physiological parameters | | |  |  |
| Weight | Normal | Mean is specific to age band and gender (overall mean for men: 82.4 kg, for women: 67.5 kg). Mean weight decreases with age. Standard deviations were calculated using a fixed assumption for the coefficient of variation (0.175 for men, 0.167 for women). This assumption was based on the analysis of patient-level data. | Source: German microcensus data. Statistisches Bundesamt (2005): Mikrozensus, Fragen zur Gesundheit - Körpermaße der Bevölkerung ***(18)*** | no |
| Blood volume | calculated | **Weight * 65** | Martinez et.al. ***(25)*** |  |
| Hemoglobin, pre-treatment | Weibull | Distribution type after analysing pre-surgery Hb values of 743 patients (see details below). Weibull is able to fit right-skewed distributions like the Hemoglobin distribution. Gender-wise different parameters: Men (location shift 4.59; shape: 8.253; scale: 10.81); Women (location shift -11.52; shape: 24.23; scale: 25.72) | Patient-level data analysis (Primary knee/hip arthroplasty data, RKU Ulm) | no |
| Hemoglobin, age correction |  | Hemoglobin level varies with age. Prevalence of anemia increases with age. Thus Hb levels were shifted upwards for patients younger than 65 years and shifted downwards for patients older than 65 years. For men, the age-specific correction of the Hb level (g/dl) ranged from -0.38 (85 years and older) to +0.06 (60-65 years) to +0.37 (up to 39 years). For women, the respective figures are -0.5 (85 years and older) to +0.06 (60-64 years) to +0.35 (up to 39 years). | Assumption backed by a variety of studies: Anemia and hemoglobin levels in older persons: relationship with age, gender, and health status. Salive ME, Cornoni-Huntley J, Guralnik JM, Phillips CL, Wallace RB, Ostfeld AM, Cohen HJ.J Am Geriatr Soc. 1992 May;40(5):489-96. See also more recent study by Tettamanti et. al. (Haematologica, 2010) | no |
| C1: Pre-surgical blood management – Treatment with Epoetin Alfa | | | | |
| Allowable Hb Range | Fixed | Pre-surgical Hb level at first visit between 10 and 13 g/dl | EPREX/ERYPO SMPC **(50)** NATA-Guidelines **(4)** | no |
| Contra- indication | Bernoulli | in 5% of all cases | Assumption | no |
| Dosage and protocol | Normal | Base case: Rosencher (2006): administration of EPO is stopped when a Hb level of 13.0 is reached (mean no. of doses: 1.9). Average increase per dose: 0.75 g/dl  (sd ± 0.58).  Alternative: Weber (2005): 4x40000 IE. Total Hb increase:  2.1 g/dl (sd ±0.07) | Rosencher ***(22)***; Weber ***(31)*** | yes: Rosencher and Weber protocols |
| Correction for Rheumatoid Arthritis | Fixed | In case of presence of RA, the effect is reduced by 0.2 g/dl. Otherwise, the effect is increased by 0.035 (in order to achieve the total EPO effect outlined above) | Reasoning based on Slappendel, Weber  ***(32)*** | no |
| C2: Pre-surgical blood management – Autologous Donation | | | | |
| Allowable Hb Range | Fixed | Pre-surgical Hb level at first visit between 11 and 13 g/dl | NATA Guidelines **(4)** | no |
| No of units collected | Fixed | 2 Units |  | no |
| Effect on pre-operative Hb level | Normal | Hb level is being lowered by PAD: Base case:  Stowell (-1.2 g/dl ± 0.87) assuming a correlation of 0.5 between the first and pre surgical measurements.  Alternative cases: Rosencher: -0.67 g/dl ± 0.88 Keating (figure 4) :  -0.8 ± 0.97 Henry (comparison 01):   -1.16 ± 0.9  Feagan: -1.05 ± 1.33 | Stowell ***(35)*** Rosencher ***(22)***  Feagan ***(34)*** Keating **(61)** Henry **(59)** | Yes: Stowell and 4 alternative scenarios |
| E: Blood loss and transfusion | | | | |
| Hemoglobin pre-surgery | Computed | **Hb pre-treatment (first visit) + EPO effect (C1)**  **+ PAD effect (C2)** |  |  |
| Transfuston  trigger | Fixed | Base case: 8.5 g/dl. Increased trigger (+1.0 g/dl) in the case of cardiac history (see phase B) |  | Alternative cases: 8.0 g/dl, 9.0 g/dl |
| Hemoglobin volume at transfusion trigger (g) | Computed | **Estimated blood volume (mL) *  Transfusion trigger (g/dL) * 100** |  |  |
| Hb loss during surgery | Normal | Base case (Pierson, 2004): Hip -4.0 g/dl (sd ±1.1 g/dl); Knee 3.8 g/dl (sd ± 1.0 g/dl). In order to achieve reasonable transfusion rates, mean values were reduced to -3.5 (hip) and -3.3 (knee). Expected blood loss has not been corrected for risk factors. | Pierson ***(45)*** Fig. 1; post hoc analysis of Weber ***(31)*** data | yes: Base case (Pierson) and three alternative settings with reduced blood loss assumptions |
| Min Hb level during surgery | Computed | Theoretical nadir without transfusion:  **Hemoglobin pre-surgery - Actual Hb loss during surgery** |  | No |
| Blood needed to meet transfusion trigger | Computed | If theoretical nadir above trigger: no transfusion Otherwise: **Estimated blood volume * ln (Hb Trigger / Hb Nadir**)  (blood volume simulated/computed in phase C) | Fomula: Waters JH, Lee SJ, Karafa MT: A Mathematical Model of Cell Salvage Efficiency. MS.Anaesthesia and Analgetisia, 2002, 95: 1312-7 | No |
| Hemoglobine level achieved by one transfusion (g/dL) | Computed | **(1 + Hemoglobin volume per unit / Hemoglobin level at transfusion trigger) * Transfusion trigger** |  | No |
|  |  |  |  |  |
| Blood loss between two transfusion (g) | Computed | **Estimated blood volume * ln (Hb level after transfusion / Hb Trigger**) | Formula: Waters JH, Lee SJ, Karafa MT: A Mathematical Model of Cell Salvage Efficiency. MS.Anaesthesia and Analgetisia, 2002, 95: 1312-7 | No |
| Units needed to maintain trigger | Computed | **(Blood needed to meet transfusion trigger) /  (Blood loss betwenn two transfusions)** rounded up to  the next whole number |  | No |
|  |  |  |  |  |
| Type of transfusion | Computed | Allogeneic. In case of pre-operative autologous donation (PAD): use pre-donated  autologous units first. If pre-donated volume of PAD is not sufficient,  use allogeneic units. |  | No |
|  |  |  |  |  |
| F: Care on ward, post-operative complications | | | | |
| Infection | Bernoulli | Infection risk based on type of transfusion: 2% if no transfusion 4% if allogeneic transfusion 6% if autologous transfusion | Freedman ***(40)***, figure 8A. Increased infection risk in case of transfusions also reported by Slappendel ***(41)*** | No |
| Length of stay (base distribution) | Extreme value | Theoretical distribution type chosen on the basis of the empirical LOS distribution for arthroplasties, RKU Ulm.  Parameters adjusted to match the mean LOS published for the patient DRG code simulated in phase A | Patient-level data analysis (Primary knee/hip arthroplasty data, RKU Ulm). Parameters adjusted to match DRG statistics published by INEK ***(26)*** | No |
| LOS increase due to allogeneic transfusion (factor) | Computed | Base case: LOS increased by 20% in case of infection  Sensitivity analysis:  Two cases with reduced effect (very low=+5%, low=+11%), two cases with increased effect (high=+29%, very high=+35%); percentages derived from regression results in Weber, Slappendel, table 4 | BEAST study (Netherlands), Slappendel ***(41)***   Weber, Slappendel ***(44)***, | Yes (base case plus 4 scenarios) |
| LOS increase due to other factors | Computed | Age Factor: mean LOS increases exponentially with age: age <= 50: factor=1 age = 60: factor=1.14 age = 70: factor=1.3 age = 80: factor=1.49  Infection: factor=1.15 Age factor and infection factor are multiplied | Weber, Slappendel ***(44)*** | No |
| Pneumonia | Bernoulli | Risk of pneumonia: 1.6% with transfusion 0.8% without transfusion | Pedersen ***(1)*** | No |
|  |  |  |  |  |
| G: Unit costs | | | | |
| EPO costs | Fixed | 800 € (4 x 40000 IE) | Janssen-Cilag average price for hospitals 2011. List price is higher. Transfusion costs are covered by the DRG and costs for EPO have to be incurred by the hospital in Germany. | Yes (Base case, 25% below, 25% above base) |
| Transfusion costs | Fixed | Allogeneic: 320 € Autologous: 250 € per unit (€) | Acquisition multiplied by 4. Shander A, Hofmann A, Ozawa S, Theusinger OM, Gombotz H et al (2010) Activity-based costs of blood transfusions in surgical patients at four hospitals. Transfusion 50(4):753-765. ***(38)*** | Yes (Base case, 25% below, 25% above base) |
|  |  |  |  |  |
| LOS cost | Fixed | 300 € per day | Institut für Entgeltwesen im Krankenhaus: DRG calculation sheets ***(26)*** | Yes (Base case, 25% below, 25% above base) |
| Pneumonia | Fixed | 5000 € per incidence | DRG-Fallpauschalenkatalog  E62B: 5,012.80 EURO |  |
| All unit costs |  |  | [www.g-drg.de](http://www.g-drg.de/)  Fallpauschalenkatalog_ 2011_100929.pdf | Yes (25% below, 25% above base) |

# Table with Sensitivity Analyses to calculate the cost delta in Euro for EPO versus No Blood Conservation and EPO versus preoperative autologous blood donation

| ***Parameter/***  ***Preoperative Hb Group [g/dl]*** | ***No Blood Conservation vs. EPO only*** | | | | | | | | ***PAD vs. EPO only*** |
| --- | --- | --- | --- | --- | --- | --- | --- | --- | --- |
|  | **10.0-10.5** | **10.5-11.0** | **11.0-11.5** | **11.5-12.0** | **12.0-12.5** | **12.5-13.0** | **10.0-13.0** | **11.0-13.0** | **11.0-13.0** |
| ***Base Case [€]*** | **509** | **372** | **286** | **162** | **67** | **16** | **113** | **84** | **859** |
| Trigger 8.0 | 447 | 276 | 125 | -4 | -79 | -115 | -26 | -60 | 729 |
| Trigger 9.0 | 482 | 391 | 374 | 321 | 240 | 191 | 262 | 247 | 973 |
| Revision patients only | 496 | 432 | 447 | 397 | 376 | 300 | 363 | 354 | 1071 |
| EPO like Weber,[[31]](http://wizfolio.com/?citation=1&ver=3&ItemID=315&UserID=2645&AccessCode=04E0840AE319464190AD9797A30C7FEA&CitationSuffix=) Trigger 8.0 | 393 | 235 | 120 | -92 | -236 | -375 | -189 | -234 | 555 |
| EPO like Weber,[[31]](http://wizfolio.com/?citation=1&ver=3&ItemID=315&UserID=2645&AccessCode=04E0840AE319464190AD9797A30C7FEA&CitationSuffix=) Trigger 8.5 | 437 | 334 | 264 | 57 | -84 | -246 | -53 | -93 | 681 |
| EPO like Weber,[[31]](http://wizfolio.com/?citation=1&ver=3&ItemID=315&UserID=2645&AccessCode=04E0840AE319464190AD9797A30C7FEA&CitationSuffix=) Trigger 9.0 | 404 | 376 | 357 | 220 | 68 | -70 | 93 | 65 | 790 |
| EPO Cost +25% | 369 | 234 | 154 | 47 | -29 | -55 | 17 | -8 | 767 |
| EPO Cost -25% | 648 | 510 | 417 | 278 | 162 | 87 | 210 | 177 | 951 |
| Transfusion Cost +25% | 642 | 484 | 371 | 223 | 107 | 42 | 162 | 127 | 909 |
| Transfusion Cost -25% | 375 | 261 | 201 | 101 | 26 | -9 | 64 | 42 | 809 |
| All Cost +25% | 636 | 465 | 357 | 203 | 83 | 20 | 142 | 106 | 1074 |
| All Cost -25% | 382 | 279 | 214 | 122 | 50 | 12 | 85 | 63 | 644 |
| Blood loss low | 110 | -37 | -230 | -292 | -305 | -261 | -251 | -276 | 469 |
| Blood loss medium | 341 | 203 | 90 | 21 | -15 | -32 | 17 | -5 | 731 |
| Blood loss base Weber et al.[[31]](http://wizfolio.com/?citation=1&ver=3&ItemID=315&UserID=2645&AccessCode=04E0840AE319464190AD9797A30C7FEA&CitationSuffix=) | 360 | 184 | -7 | -97 | -149 | -154 | -95 | -127 | 650 |
| Transfusion-related LOS increase (Lower 95% CI) | 312 | 201 | 82 | 5 | -55 | -77 | -16 | -40 | 472 |
| Transfusion-related LOS increase (Upper 95% CI) | 720 | 622 | 479 | 339 | 192 | 90 | 243 | 204 | 685 |
| PAD Cost +25% |  |  | - |  |  |  |  |  | 984 |
| PAD Cost -25% |  |  | - |  |  |  |  |  | 734 |
| PAD like Feagan et al. [[59]](http://wizfolio.com/?citation=1&ver=3&ItemID=425&UserID=2645&AccessCode=0&CitationSuffix=) |  |  | - |  |  |  |  |  | 856 |
| PAD like Henry et al. [[60]](http://wizfolio.com/?citation=1&ver=3&ItemID=404&UserID=2645&AccessCode=0&CitationSuffix=) |  |  | - |  |  |  |  |  | 848 |
| PAD like Keating et al.* |  |  | - |  |  |  |  |  | 741 |
| PAD like Rosencher et al. [[22]](http://wizfolio.com/?citation=1&ver=3&ItemID=313&UserID=2645&AccessCode=6FFB8BC0D3D447F2843BA839F130AF8E&CitationSuffix=) |  |  | - |  |  |  |  |  | 688 |
| PAD like Stowell et al. [[35]](http://wizfolio.com/?citation=1&ver=3&ItemID=297&UserID=2645&AccessCode=06B99EFBBAD24843BFCE3983975F3000&CitationSuffix=) |  |  | - |  |  |  |  |  | 853 |

EPO = epoetin; LOS = Length of stay; PAD = preoperative autologous blood donation

*Keating EM, Callaghan JJ, Ranawat AS, Bhirangi K, Ranawat CS, et al. (2007) A randomized, parallel-group, open-label trial of recombinant human erythropoietin vs preoperative autologous donation in primary total joint arthroplasty: effect on postoperative vigor and handgrip strength. J Arthroplasty 22(3): 325-333.

#

# Distribution selection for the initial Hemoglobin value (pre-surgery, first visit)

A set of 745 patients (297 men, 228 women) from RKU Universitäts- und Rehabilitations­kliniken in Ulm, Germany, who underwent primary hip or knee arthroplasty in 2007/2008, was analyzed for this purpose. The mean age was 66.2 years (+- 11.2). The Hb level measured at the first pre-surgical visit has been fitted to various distributions. Two values were missing.

The distance metric used was the Anderson-Darling statistic. Candidate distributions were Weibull, Beta, Gamma, Logistic, Lognormal and several symmetric distributions (Logistic, Normal, Student’s t, Uniform; built-in fitting procedure of Oracle Crystal ball, version 11.1.)

Weibull was best in terms of the Anderson-Darling statistic (AD statistic=0.6865). According to the AD test, there was no indication that the observed values were not drawn from a Weibull distribution (p=0.082). Another suitable candidate suggested by the fitting procedure was the logistic distribution (AD value 0.9606). However, the AD test was significant in this case, mainly due to the fact that the logistic distribution failed to fit the tails properly.

Candidate distributions and test results


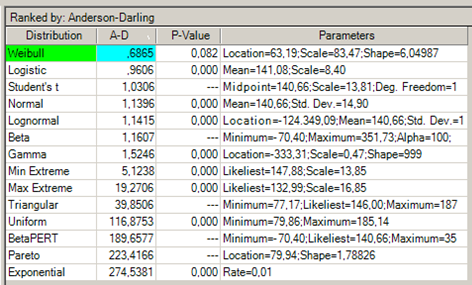


**Fit of Weibull to the observed data**


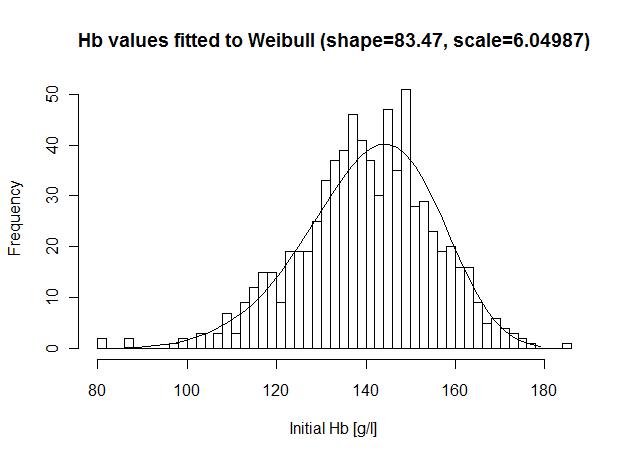


While the proportion of anemic patients (Hb < 13) was rather low in the raw data (20.5%), it is slightly higher in the fitted distribution (22.5%).
